# Biomarkers in heart failure: Traditional and emerging indicators for prognosis

**DOI:** 10.1002/ehf2.15168

**Published:** 2024-11-15

**Authors:** Arnold Péter Ráduly, László Nagy, Beáta Bódi, Zoltán Papp, Attila Borbély

**Affiliations:** ^1^ Department of Cardiology, Division of Cardiology, Faculty of Medicine University of Debrecen Debrecen Hungary

Biomarkers have long been of major importance in the diagnosis, prognosis, and follow‐up of heart failure (HF). Recently, in addition to conventional biomarkers, new types of biomarkers have emerged that are not yet used in the clinical practice and may later become of paramount importance in the long‐term management of HF. The aim of this *Editorial* is to highlight the latest advancements of HF related biomarkers that have been published in *ESC Heart Failure*.

## Introduction

Biomarkers are crucial for diagnosing, managing and predicting the outcome of HF, which is a complex condition where the heart struggles to effectively pump blood.^1^ These biological indicators encompass various proteins, enzymes and hormones present in the blood or tissue samples. They provide valuable insights into the underlying pathophysiological processes of HF, such as myocardial stress, inflammation and neurohormonal activation. By accurately reflecting disease progression and treatment response, biomarkers empower healthcare providers to make informed decisions on patient care, therapy adjustments and probable outcomes. Given the increasing prevalence of HF, biomarkers are essential for enhancing personalized medicine approaches in HF management.^2^

### Conventional biomarkers

#### NT‐proBNP

N‐terminal pro B‐type natriuretic peptide (NT‐proBNP) is one of the most prominent diagnostic biomarkers for HF, including HF with reduced ejection fraction (HFrEF), HF with mildly reduced EF (HFmrEF) and HF with preserved EF (HFpEF) as proposed by clinical guidelines.^3^ Nevertheless, NT‐proBNP plasma levels can be influenced by various factors. Although, in the presence of HF symptoms, a widely accepted recommendation^3^ sets a definite cut‐off value for the diagnosis of HF it is less clear what NT‐proBNP threshold should be considered for screening when asymptomatic, high‐risk patients. Goyder et al. analysed the results of 24 studies involving more than 26 000 patients, and they found that NT‐proBNP testing identifies left ventricular (LV) dysfunction in a high‐risk population with a sensitivity of 0.87 and a specificity of 0.84. As a result, the pooled cutoff was 311 pg/mL for NT‐proBNP and 49 pg/mL for BNP, which are considered as optimal thresholds for screening high risk and community populations.^4^ In addition, a meta‐analysis by Hendriks et al. investigated the prognostic value of BNP and NT‐proBNP levels both with and without HF across multiple prospective studies involving over thousands of patients. In their meta‐analysis, they studied the results of 66 prospective studies (38 studies with more than 46 000 patients with HF and 28 studies with more than 37 000 patients without HF) to see what predictive value they can provide for long‐term outcome and what cutoff value should be considered. The analysis found that one SD range increase of log‐transformed NT‐proBNP/BNP values were associated with a 1.7‐fold increased risk of major adverse cardiac event (MACE).^5^ Another interesting finding of this research was that NT‐proBNP/BNP can be applied for predicting cardiovascular (CV) risk of the general population with a cut‐off value of 90th percentile in healthy subjects (cut‐off values BNP/NT‐proBNP: 31.3/106 and 45.5/173 pg/mL for men and women, respectively). (As also reported in a previous study,^4^ different thresholds had to be applied when adjusted for gender.) The study conducted by Vergaro et al. demonstrated that in patients with chronic HF, gender differences were also observed in the prognostic cutoff values for NT‐proBNP, soluble suppression of tumourigenicity 2 protein (sST‐2), and high‐sensitive cardiac troponin T (hs‐cTnT).^6^ The findings indicated that women had lower cutoff values for MACE when using sST‐2 and hs‐cTnT as prognostic markers. However, NT‐proBNP had higher cutoff values for predicting 1‐year CV mortality and hospitalization in women compared to men. These results suggest the importance of considering gender‐specific thresholds when using biomarkers to predict outcomes in patients with chronic HF. Ferrannini et al. reported that natriuretic peptide levels were tested only in about half of HF patients despite an increasing trend of yearly test numbers as observed during a 7‐year‐long period starting from 2011 of the Swedish HF registry. In their analysis of over 4000 patients, natriuretic peptide levels were commonly measured in those with worsening HF symptoms, high baseline diuretic doses, elevated heart rates, or in HFpEF patients.^7^

### Sodium, potassium and chloride

Electrolyte levels are tightly controlled through complex physiological processes. Screening of plasma electrolytes is a routine and inexpensive laboratory test, commonly used not only in HF patients but also in the general patient care. Therefore, the use of electrolytes as HF biomarkers could greatly assist clinicians in their daily practice. Of note, medications commonly used in HF treatment such as those acting on the renin‐angiotensin‐aldosterone axis, sodium‐glucose‐cotransporter 2 inhibitors (SGLT2i) or various diuretics affect the body's electrolyte balance. Despite the above confounders, potassium, sodium or chloride levels can still play a prognostic role in well‐defined medical contexts during HF. For example, Xia et al. reported how different serum sodium level trajectories can affect the survival of HF patients. In their retrospective cohort study 3628 patients were included, where serial measurements on sodium level changes were conducted. The study classified participants into seven groups depending on baseline sodium levels as: hypo‐, normo‐, or hypernatremia and dynamics of sodium level changes over time. The findings indicated that rapid normalization of hyponatremia or hypernatremia was linked to an increased risk of 1‐year mortality even when accounting for all confounding factors.^8^ Sarasiti et al. reported on a study that involved a smaller group of HF patients, and focused on admission hyponatremia. Their results implicated that admission hyponatremia acts as a predictor of six‐month mortality and rehospitalization.^9^ Along this line, Lorenzo et al. performed a retrospective analysis of 874 HF patients where consecutive echocardiography was also performed, and they found that patients with high sodium levels were more prone to worsening EF during a follow up period than those who had normal sodium levels at the time of control echocardiography.^10^

Lombardi et al. analysed data from almost 1000 HF patients and investigated the prognostic significance of discharge potassium levels, specifically looking at the impact of hypo‐, normo‐, or hyperkalaemia on patient outcomes. Their study showed that hyperkalaemia at discharge is associated with higher risk of 1‐year all‐cause mortality, and thus supported awareness of potassium binders use in well‐defined HF patient groups.^11^ In contrast to the aforementioned two electrolytes, the importance of chloride ions is generally less emphasized, despite its important role in biological processes and the fact that it can be highly affected by diuretic therapy. In their recently reported prospective observational study on HFpEF patients after acute decompensation, Seo et al. provided evidence that lower chloride levels at discharge are also associated with an increased risk of mortality.^12^

### Novel biomarkers (GDF‐15, TNF‐α and antitrypsin)

Growth differentiation factor 15 (GDF‐15) is included in the transforming growth factor beta superfamily. Generally, GDF‐15 concentrations are relatively low in most organs, but its expression increases in response to injury in organs like liver, kidney or heart and could provide information about the prognosis of cardiovascular diseases. Wang et al. studied 6322 patients with stable angina and acute coronary syndrome, and they found an apparent relationship between admission GDF‐15 levels and 1‐year risk of CV death and HF events (cardiogenic shock, acute decompensation, readmission because of HF).^13^ Yin et al. sought to shed light on the prognostic value of GDF‐15 in acutely decompensated HFpEF population. The research investigated 380 HFpEF patients, and their GDF‐15 plasma levels were assessed within the first 48 h of admission. Patients belonging to higher tertiles of GDF‐15 (>4228 pg/mL) were older, with more comorbidities, more severe HF, and shorter time period between discharge and readmission than those with lower tertiles of GDF‐15 (2416–4228 pg/mL or <2416 pg/mL). The predictive power of GDF‐15 appeared to be better in 1‐year all‐cause death or HF hospitalizations than that of conventional biomarkers (NT‐proBNP, hsTnT).^14^ Zheng et al. illuminated the clinical significance of alpha‐1 antitrypsin (AAT) as biomarker in HF by using isobaric tags for relative and absolute quantitation and data processing. Circulating AAT level exhibited negative correlation with LVEF and was significantly associated with HF severity. HF linked to pulmonary hypertension carries a grim outlook, asking for reliable biomarkers for prognostic predictions.^15^ Tumour necrosis factor alpha (TNF‐alpha), an inflammatory cytokine and its related proteins, could potentially function as a new biomarker for HF associated with pulmonary hypertension. Having a non‐invasive prognostic indicator that aligns closely with outcomes holds significant value for this specific patient group. Sällberg et al. identified additional TNF‐alpha related biomarkers (LTBR, TNFRSF6B, TRAIL‐R1 and TRAIL‐R2) that could also serve as potential prognostic factors in his vulnerable patient population.^16^

Beside the aforementioned circulating novel biomarkers anti beta‐1 antibody (b1‐AAB) could modify receptor activity on cardiomyocytes. The appearance of b1‐AABs is associated with adverse prognosis; however, the role of the ABs has not been fully clarified yet. Morbach et al. investigated the prognostic effect of ABs in 47 acutely decompensated HF patients. The prevalence of ABs was near one quarter of the patients, and the presence of ABs associated with an almost 8‐fold risk for rehospitalization or CV death after a 6‐month‐long follow‐up.^17^

### General laboratory markers

Uric acid has long been known as an important marker of progressive HF, but the effects of uric acid (UA) lowering therapy on prognosis is still controversial. For this reason, Qin et al. conducted a meta‐analysis regarding the incidence, prognostic value and the influence of xanthine oxidase inhibition. Thirty‐six studies included the analysis and showed that elevated UA levels were associated with an increased risk of all‐cause mortality (near 1.5‐fold). Despite the fact, that there are novel drugs to target hyperuricemia, these drugs do not exert any effect on CV death.^18^

Decreased level of haemoglobin with or without anaemia is known as an important prognostic marker for the entire HF spectrum. However, the prognostic significance of another frequently measured haematological parameter, the mean corpuscular haemoglobin concentration (MCHC), which indirectly serves information on the carried haemoglobin has not been clarified yet. Choy et al. analysed near 1800 HFpEF patients and revealed a significant relationship between decreased mean corpuscular haemoglobin concentration (MCHC: <330 g/L) and the combined risk of CV death and HF hospitalization in patients with chronic renal failure.^19^

## Conclusion

Biomarkers have paramount importance in all aspects of HF management, as their systematic inclusion can greatly advance risk stratification and clinical decision‐making in the daily practice.

## Conflict of interest

None declared.
